# Leukotrienes in Tumor-Associated Inflammation

**DOI:** 10.3389/fphar.2020.01289

**Published:** 2020-08-19

**Authors:** Wen Tian, Xinguo Jiang, Dongeon Kim, Torrey Guan, Mark R. Nicolls, Stanley G. Rockson

**Affiliations:** ^1^Department of Medicine, VA Palo Alto Health Care System, Palo Alto, CA, United States; ^2^Department of Medicine, Stanford University School of Medicine, Stanford, CA, United States

**Keywords:** cancer, leukotrienes, inflammation, tumor microenvironment, LTB_4_

## Abstract

Leukotrienes are biologically active eicosanoid lipid mediators that originate from oxidative metabolism of arachidonic acid. Biosynthesis of leukotrienes involves a set of soluble and membrane-bound enzymes that constitute a machinery complex primarily expressed by cells of myeloid origin. Leukotrienes and their synthetic enzymes are critical immune modulators for leukocyte migration. Increased concentrations of leukotrienes are implicated in a number of inflammatory disorders. More recent work indicates that leukotrienes may also interact with a variety of tissue cells, contributing to the low-grade inflammation of cardiovascular, neurodegenerative, and metabolic conditions, as well as that of cancer. Leukotriene signaling contributes to the active tumor microenvironment, promoting tumor growth and resistance to immunotherapy. This review summarizes recent insights into the intricate roles of leukotrienes in promoting tumor growth and metastasis through shaping the tumor microenvironment. The emerging possibilities for pharmacological targeting of leukotriene signaling in tumor metastasis are considered.

## Introduction

Low-grade inflammation and dysregulated immune responses are components of the tumor microenvironment (TME), pivotal for tumor growth and response to immunotherapies (Binnewies et al., 2018). While therapies that target the immune system, such as checkpoint inhibition, have significantly improved cancer prognosis, not all cancer patients respond to immunomodulatory treatments. Additionally, some who respond initially may develop treatment resistance and autoimmunity (Wei et al., 2018). A better understanding of the biology of TME may improve the efficacy of immunotherapies and reduce the potential adverse side-effects.

Leukotrienes are proinflammatory lipid mediators that initiate inflammation and mount adaptive immune responses for host defense (Peters-Golden et al., 2005; Peters-Golden and Henderson, 2007). Activated leukotriene signaling is implicated in inflammatory manifestations of a variety of pathologies. Recent studies have demonstrated that leukotrienes also play crucial roles in shaping the tumor microenvironment. This review summarizes recent efforts in elucidating how leukotrienes modulate tumor pathophysiology and discuss possible means to harness leukotriene signaling pathways in cancer therapeutics.

## Leukotriene Synthesis

There are two types of leukotrienes (LTs): the dihydroxy fatty acid leukotriene B_4_ (LTB_4_) and cysteinyl-leukotrienes (CysLTs), including the fatty acid-peptide conjugate LTC_4_ and its metabolites, LTD_4_ and LTE_4_. Leukotrienes are mainly produced by myeloid cells, including, macrophages/monocytes, neutrophils, eosinophils, and mast cells (Peters-Golden et al., 2005; Peters-Golden and Henderson, 2007).

**Figure 1:**
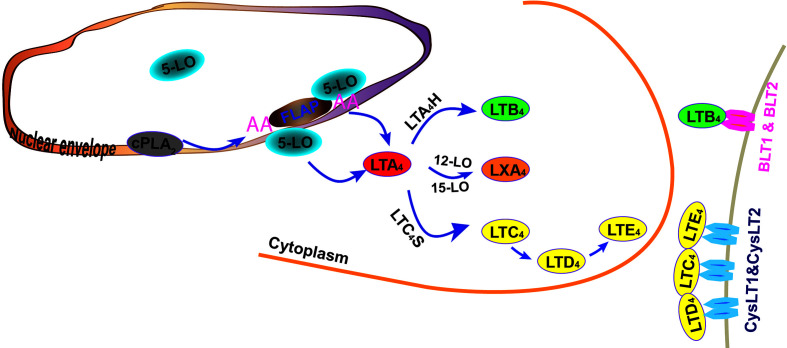
Biosynthesis of leukotrienes. Arachidonic acid (AA) is first generated by cytoplasmic phospholipase A2 (cPLA_2_) and converted into leukotriene A_4_ (LTA_4_) through the cooperative actions of 5-lipoxygenase (5-LO) and 5-LO–activating protein (FLAP). From the inner nuclear membrane, AA is metabolized into LTA_4_ by inner nuclear membrane-localized 5-LO and is further converted into LTB_4_ by LTA_4_ hydrolase (LTA_4_H). AA is also metabolized to LTA_4_ by 5-LO located on the outer nuclear membrane and converted to LTC_4_. LTC_4_S (LTC_4_ synthase) conjugates LTA_4_ with glutathione to form LTC_4_ and its metabolites, LTD_4_ and LTE_4_. (LTA_4_ may also be metabolized into lipoxin A_4_ [LXA_4_] by 12-LO or 15-LO.) Leukotrienes signal through two sets of G-protein coupled receptors, which are receptors for LTB_4_ (BLT1 and BLT2) and receptors for LTC_4_, LTD_4,_ and LTE_4_ (CysLT1 and CysLT2).

Leukotrienes biosynthesis starts from the production of polyunsaturated arachidonic acid (AA) from membrane phospholipids by phospholipase A2 (PLA2)s, especially the cytosolic form, PLA2α (cPLA2α) (Haeggstrom and Funk, 2011). 5-lipoxygenase (5-LO) is the most critical enzyme for leukotrienes production, which requires a set of stimulatory factors for its full activation, including 5-LO–activating protein (FLAP) and coactosin-like protein (Wan et al., 2017). Arachidonic acid is subsequently converted into leukotrienes in a concerted three-step reaction: first, AA is dioxygenated into 5-hydroperoxy-6-trans-8,11,14-cis-eicosatetraenoic acid [5(S)-HpETE]; second, 5(S)-HpETE is dehydrated to yield the transient epoxide intermediate, LTA_4_; and lastly, depending on the presence and functional coupling of 5-LO to its downstream enzymes, LTA_4_ is further converted to LTB_4_ by LTA_4_ hydrolase (LTA_4_H), or LTC_4_ by LTC_4_ synthase (LTC_4_S), which conjugates LTA_4_ with glutathione (Peters-Golden and Henderson, 2007; Haeggstrom and Funk, 2011; Wan et al., 2017) (**Figure 1**). Unlike many other enzymes, the catalytic activity of 5-LO and its contribution to inflammatory responses depend on its subcellular compartmentalization, phosphorylation state, and proximity to other eicosanoid-forming enzymes (Peters-Golden and Brock, 2001; Luo et al., 2003; Radmark and Samuelsson, 2009). Nuclear localized 5-LO translocates to the inner nuclear envelope and endoplasmic reticulum (ER)/Golgi membrane to facilitate the biosynthesis of LTB_4_. 5-LO from the cytoplasm favors the production of CysLT and the anti-inflammatory eicosanoid lipid, such as lipoxin A_4_ (LXA_4_) (Peters-Golden and Brock, 2001; Luo et al., 2003; Mandal et al., 2008; Radmark and Samuelsson, 2009; Haeggstrom and Funk, 2011). Non-leukocyte cells generally do not contain the full spectrum of the synthetic enzymes of leukotrienes. However, these cells, including vascular endothelial cells, express LTA_4_H and may convert LTA_4_, generated by neighboring immune cells, to LTB_4_, a mechanism known as transcellular LT synthesis (Gijon et al., 2007; Sala et al., 2010).

Leukotrienes signal through two sets of G-protein coupled receptors (GPCRs), with BLT1 (high affinity) and BLT2 (low affinity) serving as receptors for LTB_4_, and CysLT1, CysLT2, and CysLTE (also known as gpr99) as receptors for CysLTs (Peters-Golden and Henderson, 2007; Nakamura and Shimizu, 2011). Leukotrienes act in a paracrine and cell type-dependent manner, exerting their functions at nanomolar concentrations (Peters-Golden and Henderson, 2007).

## Leukotrienes and Inflammatory Disorders

LTB_4_ is noted to play essential roles in a variety of acute and chronic inflammatory diseases, including diabetes, obesity, Alzheimer’s disease, myocardial infarction, asthma, idiopathic lung fibrosis, chronic obstructive pulmonary disease (COPD), pulmonary arterial hypertension (PAH), lymphedema, and cancer (Back, 2009; Drakatos et al., 2009; Izumo et al., 2009; Wang and Dubois, 2010; Price et al., 2011; Tian et al., 2013; Li et al., 2015; Qian et al., 2015; Wculek and Malanchi, 2015; Tian et al., 2017; Michael et al., 2019; Tian et al., 2019). Mechanisms of actions of LTB_4_ in these conditions include chemotaxis for immune cell populations, facilitating endothelial adherence, and induction of blood and lymphatic vascular endothelial injury (Back, 2009; Drakatos et al., 2009; Izumo et al., 2009; Wang and Dubois, 2010; Price et al., 2011; Tian et al., 2013; Li et al., 2015; Qian et al., 2015; Wculek and Malanchi, 2015; Tian et al., 2017; Michael et al., 2019; Tian et al., 2019). LTB_4_ is one of the most powerful identified chemotactic molecules (Subramanian et al., 2017). LTB_4_ not only summons neutrophils, macrophages, mast cells, eosinophils, dendritic cells (DCs), T cells, and B cells to the site of tissue injury but also promotes the survival of these immune cells in lymphoid organs and peripheral tissues (Del Prete et al., 2007; Peters-Golden and Henderson, 2007; Subramanian et al., 2017). CysLTs is endowed with overlapping but distinct function in promoting inflammation responses when compared with LTB_4_. The classical properties of CysLTs in the vasculature, LTD_4_ in particular, are the regulation of smooth muscle contraction in the microcirculation and respiratory tract (Peters-Golden and Henderson, 2007; Araujo et al., 2018). CysLTs also induce pathological angiogenesis, maladaptive proliferative responses, expression of adhesion molecules (I-CAM-1 and VCAM-1), and loss of endothelial barrier function (Sjostrom et al., 2003; Uzonyi et al., 2006; Moos et al., 2008; Duah et al., 2013).

In the lung, 5-LO and LTB_4_ are elevated in the airways of asthma and COPD patients, as well as in the pulmonary arterioles of PAH patients, where the concentrations of LTB_4_ correlate with the severity of the disease. The upregulation of LTB_4_ and subsequent interaction with its receptors stimulate the migration of immune cells into the lungs (Drakatos et al., 2009; Tian et al., 2013). Specifically, LTB_4_ promotes the migration of immature and mature DCs along the gradient of CCL19 and CCL21 by increasing the membrane expression of CCR7 (Del Prete et al., 2007). LTB_4_ induces the rapid integrin-mediated arrest of rolling of effector and memory CD8^+^ cells and thereby mediates cytotoxic T cell trafficking (Goodarzi et al., 2003). Mast cells may secrete LTB_4_ and recruit T cells in response to tissue inflammation and infection, including allergy, asthma, and rheumatoid arthritis (Goodarzi et al., 2003). Mast cells and macrophages promote the migration of neutrophils in the presence of LTB_4_. Notably, 5-LO and LTB_4_ also interact with a number of pulmonary proinflammatory signaling molecules, including NF-κB, TNF-α, MAPK, and IL-6/STAT3, suggesting that 5-LO/LTB_4_ may further amplify the proinflammatory circuits. As an example, mice lacking 5-LO or BLT1 are impaired in TLR (toll-like receptor)-mediated NF-κB activation in pulmonary macrophages (Serezani et al., 2011).

In addition to being immunomodulatory in the lung, recent efforts also suggest that 5-LO and LTB_4_ exert key roles in the pulmonary vasculature: LTB_4_ promotes the growth and activation of pulmonary arterial smooth muscle cell (SMC) and adventitial fibroblasts. 5-LO and LTB_4_ also induce early death of pulmonary arterial endothelial cells (ECs), cause the survival cells to become apoptosis-resistant and proliferative, change the properties of these ECs to acquire endogenous 5-LO expression, and transform them into proinflammatory phenotypes; responses are implicated in the PAH pathogenesis (Tian et al., 2013; Qian et al., 2015; Tian et al., 2019). Prominent expression of LTB_4_ in the brain, adrenals, heart, adipose tissues, skin, as well as, in lymph fluid suggests an additional, less well-recognized function of this eicosanoid lipid. Emerging evidence indicates a pivotal role of LTB_4_ in insulin resistance and hepatic steatosis, with LTB_4_ directly enhancing macrophage chemotaxis, reducing insulin-stimulated glucose uptake in myocytes, and inhibiting insulin-mediated suppression of hepatic glucose output (Li et al., 2015). LTB_4_ induces the migration and proliferation of coronary artery SMCs, and BLT1 is highly expressed in the human carotid artery atherosclerotic plaques (Back, 2009). Expression of 5-LO is significantly elevated in post-mortem brain tissues of neurodegenerative diseases; also, blocking 5-LO is beneficial to aged transgenic mice with pre-existing behavioral abnormality and tau neuropathology (Tian et al., 2017; Giannopoulos et al., 2019). 5-LO activation and increased LTB_4_ concentrations are found in animal model and human lymphedema (Tian et al., 2017). LTB_4_, at low physiological concentrations, is required for lymphangiogenesis and wound healing (Tian et al., 2017; Ramalho et al., 2018). Conversely, LTB_4_, in high pathological concentrations, interferes with the protective VEGFR3 and Notch signaling in lymphatic endothelial cells, causing lymphatic vascular damage, abnormal lymphatic drainage, and lymphedema (Tian et al., 2017). The pivotal roles of LTB_4_ signaling in lymphedema are further supported in two Phase II clinical studies, where pharmacological blockage of LTB_4_ signaling is beneficial in restoring a failing lymphatic circulation (Rockson et al., 2018).

Cysteinyl leukotriene production has been directly linked to the pathogenesis of asthma (Peters-Golden and Henderson, 2007; Price et al., 2011). Extensive evidence shows that CysLTs regulate most of the key features of asthma, including airway SMC constriction, increased microvascular permeability, compromised respiratory cilia activities, airway remodeling, bronchial hyperresponsiveness, and maladaptive survival of immune cells (Price et al., 2011). Importantly, three selective antagonists of CysLT receptors (i.e., pranlukast, montelukast, zafirlukast) and the inhibitor of 5-LO (zileuton, zyflo^R^) have been used in the clinical management of asthma for nearly 20 years (Price et al., 2011). These drugs are well-tolerated and efficient in restricting bronchoconstrictor challenge mediated by allergen and exercise. CysLTs are also key mediators in allergic rhinitis, acting on nasal vascular ECs, enhancing DC-stimulated antigen presentation, activating interstitial cells (eosinophils, mast cells, macrophages, and neutrophils), and modulating nasal allergic inflammation and clinical symptoms (Sousa et al., 2002).

## Inflammation in Cancer

**Figure 2:**
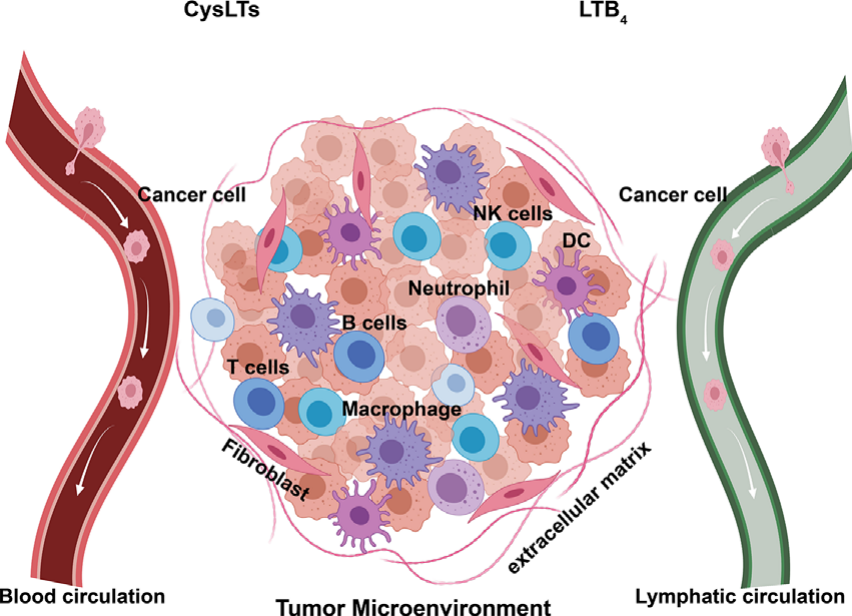
Process of cancer metastasis. 1) Genetic risks and environmental factors (e.g., inflammation) cause epithelial cell transform into cancer cell phenotype. 2) Cancer cells and cancer stem cells proliferate to generate primary tumor. 3) The carcinoma cells recruit a variety of stromal cells and immune cells to form tumor microenvironment (TME). 4) Cancer cells, under the influence of TME, acquire an invasive phenotype through epithelial-to-mesenchymal-transition (EMT) and intravasate into blood vascular or lymphatic circulation. 5) The malignant cancer cells exit the circulation and develop into secondary tumor at the distant organs.

Chronic inflammation is a significant risk factor for the development of cancer (Coussens and Werb, 2002). Immune cells, orchestrating with cancer cells and surrounding stromal cells, form an inflammatory TME (Wang et al., 2017). Cells within the TME are highly plastic. Inflammation may shape the TME towards a more malignant state and direct tumor-promoting signals (**Figure 2**). Recent transcriptomic and metabolic studies of the TME indicate enhanced expression of inflammatory cytokines and chemokines in primary tumors and metastatic lesions, which is correlated with an increased number of inflammatory infiltrates and poor clinical prognosis (Galon et al., 2006; Zhang et al., 2020). Neutralization or genetic silencing of inflammatory signaling in preclinical models diminishes tumor growth and progression. As an example, colitis-associated cancer is associated with IL-23 producing myeloid cells and IL-23-dependent TME (McGovern and Powrie, 2007). This review focuses on discussing the tumor-promoting functionality of inflammation.

### Inflammation and Tumor Initiation

Over 15% of cancers are predisposed by infection, chronic inflammation, and autoimmunity (Abu-Shakra et al., 2001; Greten and Grivennikov, 2019; Valencia et al., 2019). The most prominent examples include inflammatory bowel disease that may risk patients for colon cancer, and chronic hepatitis increases the likelihood of developing liver cancer (Uko et al., 2012). A variety of proinflammatory environmental cues may prompt cancer development, including inhalation of fine particles and tobacco smoke. Obesity, hyperglycemia, and excessive lipid accumulation promote low-grade inflammation and increase the risk of liver, pancreatic, colon, breast cancer, and other malignancies (Abu-Shakra et al., 2001; Pietrzyk et al., 2015; Greten and Grivennikov, 2019; Valencia et al., 2019). Type II diabetes is increasingly recognized as a risk factor for cancer, promoting tumorigenesis through obesity-induced inflammation and obesity-related tissue injury (Giovannucci et al., 2010).

Two interdependent events are postulated to be required for tumor initiation: 1) genetic and epigenetic alterations of tumor-suppressive pathways and oncogenic signaling, and 2) generation and growth of transformed cell clones. Inflammation, driven by macrophages and neutrophils, potently produces reactive oxygen species, which may induce gene variants (Mittal et al., 2014). Inflammation may cause germline and somatic mutations in *Tp53* and other cancer-related genes and increase the tumor mutational burden (Werner et al., 2020). IL-22, IL-6, TNF-α, and IL-1β not only cause DNA damage but also activate epigenetic machinery in epithelial cells (Grivennikov et al., 2010). Inflammatory responses may trigger the de-differentiation of epithelial cells into tumor-initiating stem cell-like cells (Sainz et al., 2016; Ayob and Ramasamy, 2018). Notably, NF-κB and IL-6/STAT3 signaling increase the survival and proliferation of the transformed cells, so-called inflammation-driven cell survival (Grivennikov et al., 2010; Chaturvedi et al., 2011; Kumari et al., 2016). Evident in liver and skin cancers, inflammation-induced cell death is required for the growth of neighboring transformed tumor cells (i.e., autophagy) (Yun and Lee, 2018). While inflammation in early-stage tumors is localized, systemic inflammation prevails during the late stage of tumor invasion and magnify the cancer sequela, as exemplified by tobacco-smoke and obesity activating neutrophils to promote breast cancer metastasis into the lungs (Walser et al., 2008; Yu et al., 2018).

### Inflammation and Tumor Progression

In a fashion similar to tumor initiation, inflammation provides direct growth signaling for tumor proliferation (Coussens and Werb, 2002). Additionally, inflammatory mediators may induce tumor cell plasticity within the TME by antagonizing potential anti-tumor immunity, stimulating angiogenesis, and recruiting fibroblasts and other stromal cells to support tumor metastasis. Furthermore, inflammatory molecules modify stromal and tumor cells metabolism and tissue stiffness by regulating the formation of extracellular matrix (Chaturvedi et al., 2011). For instance, IL-6, IL-17, and IL-11 may increase the proliferation of tumor cells, under conditions, such as chronic hypoxia, lack of nutrients, or insufficiency of anti-tumor immunity (Muz et al., 2015; Kumari et al., 2016; Zhong et al., 2016; McGeachy et al., 2019). IL-8 has been shown to recruit macrophages and neutrophils to the TME and to stimulate the angiogenic responses of vascular ECs in a paracrine manner. IL-8 may also induce carcinoma cells to acquire a mesenchymal-like phenotype (i.e., epithelial-to-mesenchymal transition, EMT) (Waugh and Wilson, 2008).

### Inflammation and Tumor Metastasis

Majority of tumor-related death is due to cancer metastasis. Growing number of studies support the role of inflammation in cancer mortality (Coussens and Werb, 2002). The migration of primary cancer cells away from the epithelium into the neighboring tissues depends on EMT (Brabletz et al., 2018; Pastushenko and Blanpain, 2019). EMT enhances the mobility of cancer cells and allows them to break from the basal membrane and enter into lymphatic and blood circulation for further dissemination (Brabletz et al., 2018; Pastushenko and Blanpain, 2019). Cancer stem cells adapt transcriptional and functional similarities to mesenchymal cells in motility (Brabletz et al., 2018; Pastushenko and Blanpain, 2019). TNF-α and IL-1β directly induce the expression of key transcription factors for EMT, SLUG, SNAIL, and Twist (Brabletz et al., 2018; Pastushenko and Blanpain, 2019). IL-11 is associated with the recruitment of fibroblasts, supporting tumor invasion, immune escape, and selection of malignant cancerous cells; increased IL-11 expression is correlated with worse clinical prognosis (Zhong et al., 2016). IL-17 activates neutrophils and drives breast cancer metastasis by facilitating the formation of a pre-metastatic niche (Gu et al., 2015). Inflammatory signals also prompt the expression of tissue-specific adhesion molecules and integrins, thereby aid the generation of tropism of metastasis (Bendas and Borsig, 2012). In malignant tissue, tumor-associated macrophages (TAMs) are the most abundant cell types of TME; these polarized macrophages mediate tumor growth and angiogenesis, secrete pro-tumor signaling molecules, and suppressing anti-tumor adaptive immune responses (Noy and Pollard, 2014; Che et al., 2017).

### Therapy-Induced Inflammation in Cancer

Chemotherapy, radiotherapy, and surgical interventions may generate inflammatory responses as well. Damage-associated molecular patterns (DAMPs) released from dying tumor cells may regulate the synthesis of IL-1β and other cytokines; DAMPs also induce and sustain *de novo* anti-tumor T cell responses (Hernandez et al., 2016). In contrast, dying tumor cells can stimulate the production of TNF-α, epidermal growth factor (EGF), IL-6, and Wnt ligands, which in turn recruit myeloid cells and fibroblasts to the local TME, serving as anti-cell death signals and decreasing the efficacy of anti-tumor therapies (Labi and Erlacher, 2015). For example, paracrine EGFs, which are secreted from macrophages or fibroblasts, are major factors causing cancer therapy resistance (Srivatsa et al., 2017).

## Leukotrienes in Cancer

**Figure 3:**
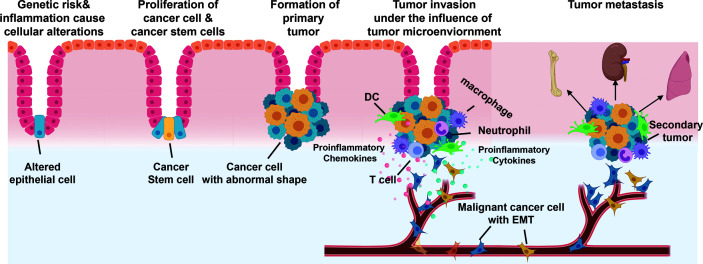
Components of tumor microenvironment (TME). Established primary tumor consists of a wide array of cellular infiltrates, including immune cells of both innate and adaptive immunity. These cells secrete proinflammatory cytokines, chemokines, and leukotrienes, as well as form a complex regulatory network that fosters tumor metastasis by creating an environment enabling cancer to evade immune surveillance and destruction. DC, dendritic cells; NK cells, natural killing cells; CysLTs, LTC_4_, LTD_4,_ and LTE_4_.

Elevated 5-LO and leukotriene signaling is reported in various forms of cancers, including cancer of the pancreas, colon, stomach, prostate, ovaries, and lungs (Wang and Dubois, 2010; Jala et al., 2017). These proinflammatory mediators may modulate the initiation, progression, and metastasis of tumors through regulating the proliferation, apoptosis, migration, and invasion of cancer cells. 5-LO and leukotriene signaling may also be capable of shaping the TME through inducing the migration and activation of immune cells, production of growth factors, secretion of proinflammatory mediators and angiogenic factors; they may also directly interact with blood and lymphatic endothelium and influence the migration of cancer cells (**Figure 3**). The remainder of this review discusses the specific links between 5-LO and leukotriene-mediated signaling in cancer by summarizing and speculating the roles of 5-LO and leukotriene in promoting tumorigenesis and tumor microenvironment.

### 5-LO and Leukotriene Signaling and Cell Growth

Western/Northern blotting and histology are not able to detect 5-LO expression in healthy pulmonary ECs (Walker et al., 2002; Zhang et al., 2002; Porter et al., 2014). However, antagonizing 5-LO signaling arrests ECs from dividing in culture and chronic hypoxia induces abnormal EC proliferation in a 5-LO-dependent fashion, implicating that 5-LO expression is required for EC mitosis (Walker et al., 2002; Porter et al., 2014). Additionally, 5-LO is expressed in the nuclei of a number of cancer stem cells; 5-LO causes ECs lack of BMPR2 signaling to transform into a cancer stem cell-like phenotype, and targeting 5-LO suppresses the adverse growth responses (Romano et al., 2001; Chen et al., 2009a; Chen et al., 2009b; Roos et al., 2014; Tian et al., 2019). The mechanisms by which 5-LO signaling regulate cell proliferation is not understood but is postulated to be related to the nuclear localization of this eicosanoid enzyme. Consistently, inhibition of LTA_4_H impedes the growth of colon cancer cells, suggesting that LTB_4_ is essential for cancer cell growth (Jeong et al., 2009; Lin et al., 2016; Zhao et al., 2019). LTB_4_ directly stimulates colon cancer cell growth and survival through a BLT1/ERK-dependent pathway *in vitro*; it induces the proliferation of human pancreatic cancer cells through MAPK/ERK and PI3K/Akt-dependent pathways; both LTB_4_ and CysLTs prompt the expansion of CD24^+^CD90^+^ metastasis-initiating cells (Tong et al., 2005; Ihara et al., 2007; Wculek and Malanchi, 2015). Activation of LTD_4_ promotes the growth and survival of human intestinal epithelial cells through multiple parallel pathways, including GSK3β/β-catenin, PKC/Raf/ERK1, and ERK2 signaling (Paruchuri et al., 2002; Mezhybovska et al., 2006). By contrast, inhibition of LTD_4_ signaling by a CysLT1 antagonist causes apoptosis of prostate carcinoma cells (Matsuyama et al., 2007; Larre et al., 2008). Increased CysLT1 and CysLT2 expression are associated with LTD_4_-induced apoptosis-resistance, hyper-proliferation of colorectal cancer cells, and poor clinical prognosis (Magnusson et al., 2007; Jeong et al., 2009). A prominent role of leukotrienes in the regulation of stem cell homeostasis is established, with LTB_4_ and LTD_4_ known to stimulate the proliferation and maturation of several types of stem cells, progenitor cells, and cancer stem cells (Chung et al., 2005; Paruchuri et al., 2006; Wada et al., 2006).

### Leukotrienes and Epithelial (Endothelial)-to-Mesenchymal Transition

Alterations in cell phenotypes, EMT in particular, have been shown to play an important role in tumorigenic processes (Brabletz et al., 2018; Pastushenko and Blanpain, 2019). Complete and partial EMT in cancer is executed by EMT-activating transcription factors, including SNAIL, TWIST, and ZEB families, which regulate all stages of cancer progression from initiation, primary tumor growth, invasion, metastasis, to colonization, as well as resistance to therapy (Brabletz et al., 2018; Pastushenko and Blanpain, 2019). In addition, EMT-activating transcription factors have been shown to be critical for the maintenance of cancer stemness (Pastushenko and Blanpain, 2019). Specifically, studies focusing on IL-6, IL-8, and TNF-α demonstrate that expression of these inflammatory mediators is associated with clinical occurrences of EMT and resistance to EGFR inhibition (Singh et al., 2013; Thompson et al., 2015).

In culture, LTB_4_, *via* BLT2, promotes EMT and the expression of vimentin in some human cancer cell lines through activation of reactive oxygen species, NF-κB, TGF-β, and ERK (Kim et al., 2014). LTB_4_ also upregulates the production of IL-8 in breast cancer cells, while blocking BLT2 suppresses the formation of metastatic lung nodules in animal models of breast cancer metastasis (Kim et al., 2012). In coordination with NF-κB signaling, LTB_4_ mediates the synthesis of both IL-6 and IL-8 to increase the invasiveness of cancer cells (Thompson et al., 2015). Exogenous LTB_4_ or 5-LO causes pulmonary arterial EC, derived from patients with BMPR2 mutations, to undergo endothelial-to-mesenchymal-transition (EndMT) through activated TGF-β signaling; these cells lose the typical endothelial cobblestone appearance and, instead, acquires an elongated mesenchymal cell shape with increased expression of mesenchymal markers (vimentin, SM-actin, and SLUG) and inflammatory molecules (IL-6, IL-1β, and TNF-α), a process resembling EMT (Tian et al., 2019).

### Leukotrienes and Tumor Microenvironment

A growing body of literature indicates that 5-LO and leukotrienes are critical components TME, mediating the crosstalk between epithelial cells, stromal cells, and immune cells. In lung cancer, a complex molecular interaction initiated by mast cell-derived LTB_4_ has been described, in which mast cells orchestrate with tumor-promoting neutrophils through production of LTB_4_ (Malaviya and Abraham, 2000; Satpathy et al., 2015). Neutrophils support the metastatic transformation and colonization of breast cancer cells through leukotriene-mediated ERK activation (Wculek and Malanchi, 2015). Treatment with zileuton reduces systemic inflammation, blocks macrophage infiltrates, and decreases polyp burden of the small intestine and colon in a murine model of polyposis (Gounaris et al., 2015). Genetic deletion or pharmacological ablation of 5-LO or LTA_4_H significantly reduces the tumor burden in K-ras–driven pancreatic ductal adenocarcinoma and in xenograft mouse model of human pancreatic cancer, through reduction of TNF-α secretion (Oi et al., 2010; Knab et al., 2015). TNF-α, one of the main cytokines in the TME, has a context-dependent role in tumor growth (Balkwill, 2009). In a cooperative manner with TNF-α, LTB_4_ may influence the growth, survival, invasion, and metastasis of tumor cells.

### Leukotrienes and Tumor Suppression

Human and animal studies suggest a positive correlation of prolonged survival with the presence of tumor-infiltrating CD8^+^ T cells (Galon et al., 2006; Kmiecik et al., 2013). LTB_4_/BLT1 signaling is required for cytotoxic T cells accumulation during allergic inflammation (Miyahara et al., 2005). A recent study demonstrates a pivotal function of LTB_4_/BLT1 signaling for the tumor immune suppression of CD8^+^ T cells: CD8^+^ T cells depletion enhances tumor growth in wild-type but not in *BLT1^−/−^* mice, implicating the importance of BLT1 in CD8^+^ T cells cancer immunity (Sharma et al., 2013). The presence of tumor-infiltrating lymphocytes correlates with the responsiveness to PD-1 (programmed cell death protein 1)-targeting cancer therapies (Yao et al., 2018). Notably, PD-1 blockade fails to reduce melanoma growth in *BLT1^−/−^* mice due to deficiency in T cell infiltrations (Chheda et al., 2016). Collectively, these findings suggest an important role of LTB_4_ signaling to facilitate the migration of tumor-infiltrating lymphocytes in anti-tumor immunity in cancer.

### Leukotrienes in Angiogenesis and Lymphangiogenesis

To initiate metastasis, cancer cells disseminate from the primary tumor either through blood (hematogenous spread after angiogenesis) or through the lymphatic (lymphogenous spread after lymphangiogenesis) circulation (Christiansen and Detmar, 2011; Ziyad and Iruela-Arispe, 2011). It is generally believed that alterations in the primary TME and EMT of tumor cells facilitate the migration towards blood or lymphatic vessels (Christiansen and Detmar, 2011; Ziyad and Iruela-Arispe, 2011). Although not fully understood how the route of intravasation is determined (i.e., hematogenous or lymphogenous), inflammatory signaling appears to be the key regulator attracting tumor cells towards circulation (Noonan et al., 2008; Zuazo-Gaztelu and Casanovas, 2018). Blocking 5-LO or LTB_4_/BLT2 signaling significantly inhibits the VEGF-A-mediated angiogenic responses (Tsopanoglou et al., 1994). LTC_4_ and LTD_4_, working through CysLT2, enhance angiogenesis and the permeability of blood vessels, independent of VEGF-A signaling, and thereby, contribute to tumor metastasis (Kim et al., 2009). LTB_4_ poses bimodal effects on lymphatic vessel health: at low concentrations, LTB_4_ promotes lymphatic EC growth and sprouting, while high concentrations of LTB_4_ inhibit lymphangiogenesis and induce apoptosis of lymphatic ECs (Tian et al., 2017). Recent studies have suggested that a viable lymphatic vasculature promotes the efficacy of immunotherapy (Fankhauser et al., 2017; Jiang, 2020). Therefore, LTB_4_ may also influence immunotherapeutics by affecting the growth and survival of lymphatic vessels.

## Therapeutic Interventions Targeting Leukotrienes and 5-LO

Many anti-inflammatory agents, including NSAIDs (nonsteroidal anti-inflammatory drugs) which inhibits cyclooxygenase (COX-1, COX-2, or both) or both COX and 5-LO, as well as, specific inhibitors of 5-LO and leukotriene signaling, have demonstrated promising results in interfering with the tumor microenvironment (Cruz-Correa et al., 2002; Baron, 2003; Rayburn et al., 2009; Wong, 2019). In particular, a meta-analysis of 16 independent studies, including 202,780 patients with a diagnosis of prostate, lung, colorectal, and breast cancers, suggests the potential of NSAID in reducing tumor incidence and mortality (Cruz-Correa et al., 2002). Specifically, familial adenomatous polyposis (FAP) patients receiving NSAID (celecoxib or sulindac), dual blockers of COX-2 and 5-LO, display a decreased recurrence, lower polyp number, and a regression of existing adenomas (Steinbach et al., 2000; Cruz-Correa et al., 2002; Maier et al., 2008; Steinbrink et al., 2010; Lynch et al., 2016). Celecoxib was approved by FDA as adjuvant therapy for FAP in 2011. The potential usage of rofecoxib or valdecoxib, two other dual inhibitors of COX-2 and 5-LO, as adjuvant therapy for tumor metastasis, is still under investigation. Nevertheless, the application of NSAIDs as anticancer agents remains controversial because of their possible gastrointestinal and cardiovascular toxicity.

Antagonists specific for 5-LO and leukotrienes are well-tolerated and confer no adverse effect in the gastrointestinal and cardiovascular systems. Despite numerous reports of the anti-tumor properties of these agents in preclinical and cell culture studies, only a few clinical trials have been conducted to evaluate their potential in cancer treatment. The 5-LO inhibitor, zileuton, has shown positive results in treating experimental models of colon, lung, and pancreatic cancers (Rioux and Castonguay, 1998; Wenger et al., 2002; Chen et al., 2004; Wculek and Malanchi, 2015). Blocking FLAP also indicates therapeutic benefit in the hamster model of pancreatic cancer. A few clinical trials have been conducted using LY293111, a well-tolerated inhibitor of BLT1, in patients with pancreatic cancer and non-small cell lung cancer; no significant difference in short-term survival was noted from these human studies (Ding et al., 2005; Janne et al., 2014). Notably, LTA_4_H inhibitor, ubenimex, has been marketed in Japan for over 30 years as an adjunct therapy for adult acute leukemia and lung cancers.

Various natural compounds, found in food and plants, may inhibit COX-2 and 5-LO pathways. Resveratrol (enriched in grapes and red wine), ginsenosides (found in ginseng), s-allylmercaptocysteine (purified from garlic), and turmeric curcumin (present in curry) are among these categories (Das and Das, 2007; Park et al., 2007; Li et al., 2014; Jeong et al., 2016). A number of recent clinical studies demonstrate encouraging results in the usage of these natural compounds in combination with conventional cancer therapies, possibly through arresting cancer cell growth, potentiating cellular apoptosis, inhibiting NF-κB, MAPK, JNK, and VEGF pathways and sequestering reactive oxygen species (Hassan, 2004; Park et al., 2007; Hofseth and Wargovich, 2007; Howard et al., 2008).

## Concluding Remarks

Promising preclinical results have motivated the pharmaceutical industry to develop anti-5-LO and anti-leukotriene drugs to treat a wide range of cancers (Orafaie et al., 2020). To date, most of the small molecule inhibitors in these classes failed to merit clinical application. Leukotrienes are typically generated by activated leukocytes; therefore, the impact of these mediators depends on the temporal accumulation and composition of immune cells at various stages of cancer. Leukotrienes fall into a big category of eicosanoid metabolites which are interconnected in a complex manner. Modulating one pathway may likely cause shunting the eicosanoid synthesis to lipids with opposing bioaction [e.g., blocking macrophage production of LTB_4_ causes increased expression of PGE_2_, which is implicated in multiple pro-tumor responses (Nakanishi and Rosenberg, 2013)]. Inflammation is the primary risk factor for the development of certain types of cancers. The proinflammatory tumor microenvironment is pivotal to the spread of cancer. However, immunotherapies, such as checkpoint inhibition and adoptive cell transfer, may benefit from a fine-tuned and tumor-specific T cell response. Understanding the communications between cancer and inflammation will facilitate the discovery of the next breakthrough in cancer management. 5-LO and leukotriene signalling is a critical component of the inflammatory tumor microenvironment: leukotrienes are strong chemoattractants for leukocytes; 5-LO and leukotrienes may elicit robust immune reactions by promoting the growth and survival of immune cells; 5-LO and leukotrienes participate in the maladaptive growth response of cancer cells and may modulate the expansion of blood and lymphatic vessels around primary tumor (i.e., angiogenesis and lymphangiogenesis); and last but not least, interactions between 5-LO and leukotriene signaling and a number of inflammatory pathways (e.g., COX, IL-1, IL-6, IL-17, NF-κB, and TNF-α) are identified in various inflammatory conditions, including cancers. Elucidating the biology of eicosanoids in tumorigenesis, profiling these biologically active lipids and their associated enzymes and receptors in cancer, and gaining structural insight into the eicosanoid proteins are crucial steps to inform future efforts in developing biomarkers and designing drug targets.

## Author Contributions

Conceived and participated in the writing of this manuscript (WT, XJ SR). Assisted in the preparation and editing of the manuscript (DK, TG, MN).

## Funding

This work was supported by Stanford Endowed Chair funds to (MN and SR), the NIH K12 HL120001-05 (to XJ) and the NIH HL095686, HL141105, HL138473 to (MN).

## Conflict of Interest

The authors declare that the research was conducted in the absence of any commercial or financial relationships that could be construed as a potential conflict of interest.
